# Circular RNAs as Potential Theranostics in the Cardiovascular System

**DOI:** 10.1016/j.omtn.2018.09.022

**Published:** 2018-10-02

**Authors:** Yihua Bei, Tingting Yang, Lijun Wang, Paul Holvoet, Saumya Das, Joost P.G. Sluijter, Marta Chagas Monteiro, Yang Liu, Qiulian Zhou, Junjie Xiao

**Affiliations:** 1 Cardiac Regeneration and Ageing Lab, Institute of Cardiovascular Sciences, School of Life Science, Shanghai University, Shanghai 200444, China; 2 Department of Cardiovascular Sciences, Experimental Cardiology, KU Leuven, 3000 Leuven, Belgium; 3 Cardiovascular Division of the Massachusetts General Hospital and Harvard Medical School, Boston, MA 02114, USA; 4 Department of Cardiology, Laboratory of Experimental Cardiology, University Medical Center Utrecht, Utrecht 3508GA, the Netherlands; 5 UMC Utrecht Regenerative Medicine Center, University Medical Center Utrecht, Utrecht 3508GA, the Netherlands; 6 Pharmaceutical Science Post-Graduation Program, Health Science Institute, Federal University of Pará/UFPA, Belém, PA 66075900, Brazil; 7 Department of Cardiology, Tongji Hospital, Tongji University School of Medicine, Shanghai 200065, China

**Keywords:** cardiovascular disease, circular RNA, myocardial infarction, heart failure, cardiomyopathy, atherosclerosis, fibrosis, endothelial dysfunction, therapeutic targets, biomarkers

## Abstract

Cardiovascular diseases (CVDs) represent the largest contributor to mortality worldwide. Identification of novel therapeutic targets and biomarkers for CVDs is urgently needed. Circular RNAs (circRNAs) are endogenous, abundant, and stable non-coding RNAs formed by back-splicing events. Their role as regulators of gene expression has been increasingly reported. Notably, circRNAs mediate essential physiological and pathological processes in the cardiovascular system. Our first aim, therefore, is to summarize recent advances in the role of circRNAs in cardiac development as well as in pathogenesis of various CVDs. Because circRNAs are stable in circulation and their dynamic changes may reflect different disease stages, they are considered ideal biomarkers. Therefore, our second aim is to review studies that have identified circulating circRNAs as biomarkers for CVDs. Finally, we discuss the shortage of functional studies and the limitations of available clinical studies and provide future perspectives.

## Main Text

Cardiovascular diseases (CVDs) represent the largest contributor to mortality worldwide, leading to almost half of the 36 million annual deaths due to non-communicable diseases in the world.^1^ With the increasing aging population, the burden of CVDs is forecast to reach epidemic proportions.^2^ Identification of novel therapeutic targets and biomarkers for CVDs are urgently needed, which may improve current strategies for diagnosis or treatment and may also contribute to reducing the morbidity and mortality from CVDs.

Non-coding RNAs (ncRNAs) are a large group of RNAs that are thought not to code for proteins. These ncRNAs are generally divided into two categories according to their nucleotide length: small ncRNAs and long ncRNAs. MicroRNAs (miRNAs, miRs) are a group of small ncRNAs that contain 19–24 nt, whereas long ncRNAs (lncRNAs) contain >200 nt.^3^, ^4^ Although ncRNAs do not code for proteins, they play important roles in the regulation of gene expression. MiRNAs have been well known to regulate target genes by mRNA degradation or disruption of their translation initiation.^5^ Due to the versatility of RNA itself, lncRNAs can regulate gene expression through chromatin remodeling, transcriptional control, and posttranscriptional processing.^6^

In addition to linear ncRNAs, circular RNAs (circRNAs) are typically formed when the 5′ end of pre-messenger RNA (pre-mRNA) is covalently back-spliced to the 3′ end.^7^ As novel and important regulators of gene expression, circRNAs have been increasingly reported to be involved in many essential processes in the cardiovascular system.^8^, ^9^, ^10^ In this review, we summarize the roles of circRNAs in cardiac development and multiple CVDs and also describe recent findings of circulating circRNAs as potential biomarkers for CVDs.

### Biological Function of circRNAs

circRNAs, initially considered to be secondary byproducts of linear mRNA splicing, have been proved to be endogenous, abundant, stable, and functional ncRNAs in mammalian cells.^11^ According to component derivation, circRNAs are generally divided into three types: circRNA derived from exons (ecircRNA), circRNA derived from lariat introns (ciRNA), and circRNA derived from exons with retained introns (EIciRNA).^12^, ^13^, ^14^ Although the abundance of these three types of circRNAs differs across different species and tissues, it has been reported that most of the known circRNAs are derived from exons, while other types of circRNAs are taking relatively small proportions.^15^, ^16^ Compared to miRNAs and lncRNAs, the understanding of circRNA functions are still limited. circRNAs have been reported to function as competing endogenous RNA or miRNA sponges, modulate the stability of mRNA, interact with RNA-binding proteins (RBPs), and regulate gene transcription and mRNA translation.^11^, ^17^

circRNA ciRS-7 (circRNA sponge for miR-7), also known as Cdr1as, is a well-studied endogenous miRNA sponge that could associate with Argonaute 2 (Ago2) in a miR-7-dependent manner and strongly suppress miR-7 activity.^18^ CircHIPK3 has also been reported to directly bind to miR-124 and inhibit its activity.^19^ In heart, heart-related circRNA (HRCR) could act as a sponge for miR-223 and protect against cardiac hypertrophy and heart failure (HF) by inhibiting miR-223 activity.^20^

In addition to their role as miRNA sponges, many circRNAs are predicted to bind to RBP and some of them have been reported to interact with RBPs.^17^ CircFOXO3 could form ternary complex with the cell-cycle proteins cyclin-dependent kinase 2 and cyclin-dependent kinase inhibitor p21 and thus block cell-cycle progression.^21^ In heart, circFOXO3 has high binding affinity to senescence and stress-related factors, which may promote cardiac senescence.^22^

Multiple studies show that circRNAs have the ability to regulate gene transcription. Exon-intron circRNAs such as circEIF3J and circPAIP2 could bind to RNA polymerase II (Pol II) and regulate the expression level of their host genes.^14^ Although circRNAs are identified as a class of ncRNAs, some of them have been reported to be translated.^23^, ^24^ CircZNF609 contains an open reading frame driven by N6-methyladenosine and can be translated into a specific protein in a cap-independent manner.^25^ CircFBXW7 driven by internal ribosome entry site (IRES) encodes a novel protein that could inhibit glioma tumorigenesis.^26^

Recently, Cdr1as knockout mice have been successfully generated using CRISPR-Cas9 and display-impaired sensorimotor gating and dysfunctional synaptic transmission.^27^ Interestingly, Cdr1as knockout mice had reduced expression of miR-7 in brain tissue, since Cdr1as was supposed to regulate miR-7 stability at posttranscriptional levels.^27^ In contrast, miR-671 has a perfect complementary binding site with Cdr1as, which may cause slicing of Cdr1as and release of miR-7 cargos.^27^ This is the first real *in vivo* evidence that a circRNA has a biological function and may discover a novel mode of action between circRNA and miRNA.^28^

**Figure 1:**
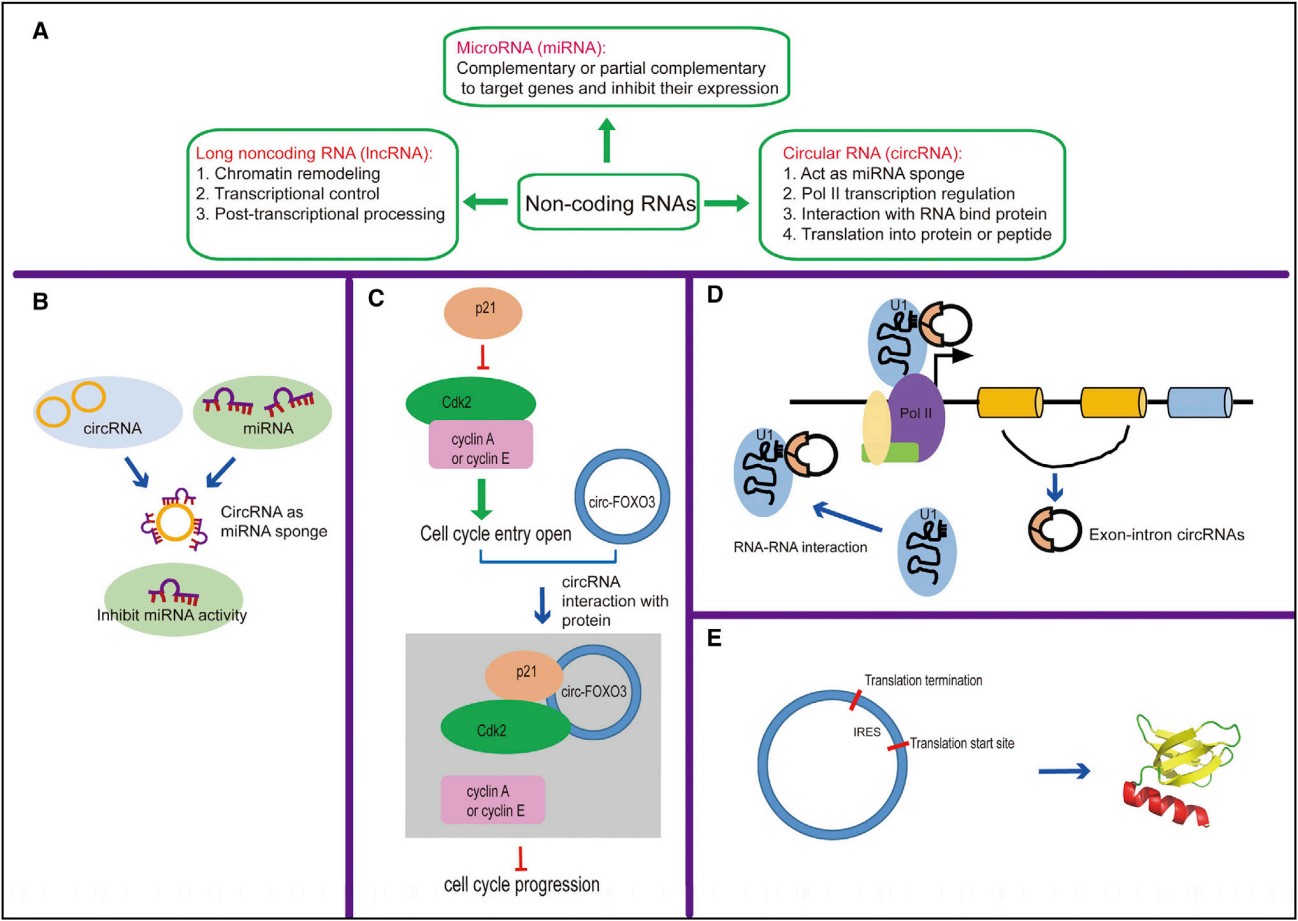
Biological Functions of Circular RNAs (A) An overview of biological functions of microRNA (miRNA), long non-coding RNA (lncRNA), and circular RNA (circRNA). (B) circRNAs could function as competing endogenous RNAs or microRNA sponges. (C) An example of circRNAs that could interact with RNA-binding proteins (RBPs): circ-FOXO3 could form ternary complex with the cell-cycle proteins cyclin-dependent kinase 2 (CDK2) and cyclin-dependent kinase inhibitor 1 (p21) and thus repress cell-cycle progression. (D) circRNAs could bind to RNA polymerase II (Pol II) and regulate the transcription of their host genes. (E) Some of the circRNAs have internal ribosome entry sites (IRESs) and could be translated.

Indeed, circRNAs have diverse modes of action (Figure 1). However, it should be noted that much of our understanding comes from a small number of circRNAs, and it is unclear whether these are representative of the group as whole. For a better study of circRNAs, several online databases have been developed that could be useful for prediction of possible interaction for circRNAs of interest and for association of circRNAs with diseases^29^, ^30^, ^31^, ^32^, ^33^, ^34^, ^35^, ^36^, ^37^, ^38^, ^39^ (Table 1). Further in-depth investigation of the biological functions of circRNAs is needed.

**Table 1:** List of Online Databases Associated with circRNAs

Database	Description	Website	Reference
circBase	designed for users to collect, unify, and annotate circRNA data and provides custom python scripts to identify known and novel circRNAs in sequencing data	http://www.circbase.org	^29^
starBase	designed for systematically identifying the RNA-RNA and protein-RNA interaction networks from 108 CLIP-Seq datasets	http://starbase.sysu.edu.cn	^30^
circRNADb	a comprehensive database for human circRNAs with protein-coding annotations	http://202.195.183.4:8000/circrnadb/circRNADb.php	^31^
CircInteractome	a website for exploring circRNAs and their interacting proteins and miRNAs	https://circinteractome.nia.nih.gov	^32^
CIRCpedia v2	contains circRNA annotations and allows users to search, browse, and download circRNAs with expression characteristics in various cell types/tissues	http://www.picb.ac.cn/rnomics/circpedia	^33^
deepBase	designed for identification, expression, evolution, and function of small RNAs, lncRNAs, and circRNAs from deep-sequencing data	http://rna.sysu.edu.cn/deepBase	^34^
circlncRNAnet	an integrated web-based resource for obtaining multiple lines of functionally relevant information on circRNAs/lncRNAs of their interest	http://app.cgu.edu.tw/circlnc	^35^
CSCD	a database designed for cancer-specific circRNAs	http://gb.whu.edu.cn/CSCD	^36^
exoRBase	a database providing information about circRNA, lncRNA, and mRNA in human blood exosomes	http://www.exoRBase.org	^37^
circRNADisease	a database providing experimentally supported circRNA-disease associations	http://cgga.org.cn:9091/circRNADisease	^38^
Circ2Disease	a database for experimentally supported associations between circRNAs and diseases	http://bioinfo.snnu.edu.cn/circr2disease/	^39^

### circRNAs in the Heart

A number of the 575 candidate circRNAs passed a test for enrichment in RNase R-treated samples in comparison to untreated ones using circTest in adult mouse heart.^40^ Many of these circRNAs were found to correspond to the genes that have been previously linked to CVDs, such as Ryr2, Hectd1, and Ppp2r3a.^40^ To further characterize circRNAs in heart, nuclear and cytoplasmic RNAs were separated, and RNA co-immunoprecipitated with endogenous Ago2 was analyzed in neonatal rat cardiomyocytes (CMs).^16^ circRNAs were found to be highly enriched in the cytoplasm compared to linear transcripts.^16^ Moreover, circRNAs had a similar level of association with Ago2, indicating that circRNAs and linear transcripts may have similar capacity to interact with miRNAs.^16^

More recently, based on deep RNA-sequencing on ribosomal-depleted RNA and purpose-designed bioinformatics tools, a total of 15,318 and 3,017 cardiac circRNAs were identified, respectively, in human and mouse heart, with a majority derived from exons and largely spliced from coding exons (CDS).^41^ Interestingly, top-expressed circRNAs in the human heart were generated from the muscle (cardiac and/or skeletal)-expressed genes, including TTN, RYR2, and DMD.^41^ Moreover, circSLC8A1-1 (a single-exon circular isoform from the Na^+^/Ca^2+^ exchanger gene SLC8A1) was identified as the most abundant circRNA in the human heart; circSlc8a1-1 generating from the same exon in SLC8A1 also ranked as the second most abundant circRNA in the mouse heart.^41^ The candidate circRNAs identified in the heart and their association with host genes may suggest their potential role in cardiac physiology and diseases, which need more functional investigation.

### circRNAs in Cardiac Development

**Figure 2:**
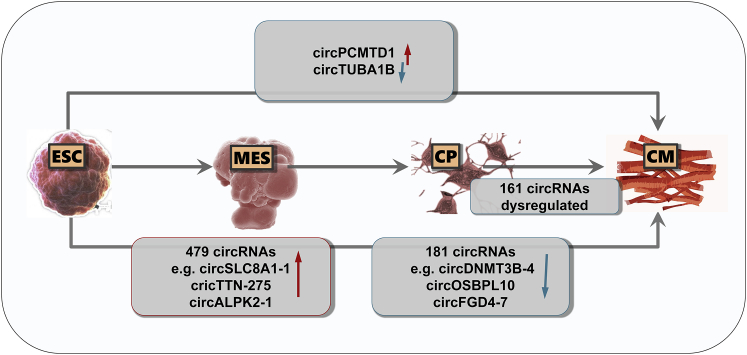
Circular RNAs in Cardiac Development

The roles of circRNAs in cardiac development are summarized in Figure 2. The TTN gene can undergo a complex alternative splicing during cardiac maturation and dozens of circRNAs arise from it.^16^ TTN-derived circRNAs were differentially expressed in neonatal and adult rat hearts, suggesting that they may participate in postnatal heart growth.^16^ The expression of lncRNAs, circRNAs, and protein-coding genes was determined by integrating RNA-sequencing data at different differentiation stages based on human embryonic stem cells (hESCs), including undifferentiated (ESC), mesoderm (MES), cardiac progenitor (CP), and definitive CM.^42^ A total of 161 circRNAs were differentially expressed during the differentiation from CP to CM, and most of them were increased in CM, suggesting a critical role of circRNAs in cardiac cell specification.^42^ Interestingly, circPCMTD1 continuously increased while circTUBA1B continuously decreased during all differentiation stages.^42^ Functional enrichment analysis further predicted the biological pathways that may be regulated by circRNAs and suggested their association with cardiac-cell specification and differentiation.^42^ In addition, 479 circRNAs were found to have a strong positive correlation with the differentiation time course of hESC to CM, which included circSLC8A1-1, circTTN-275, and circALPK2-1.^41^ In contrast, 181 circRNAs were found to have obvious negative correlation with the differentiation time course of hESC to CMs, which included circDNMT3B-4, circOSBPL10, and circFGD4-7.^41^ However, these studies are mostly descriptive and not *in vivo*; thus, functional studies are highly needed in the future to clarify the role of circRNAs in cardiac development.

### circRNAs in CVDs

**Figure 3:**
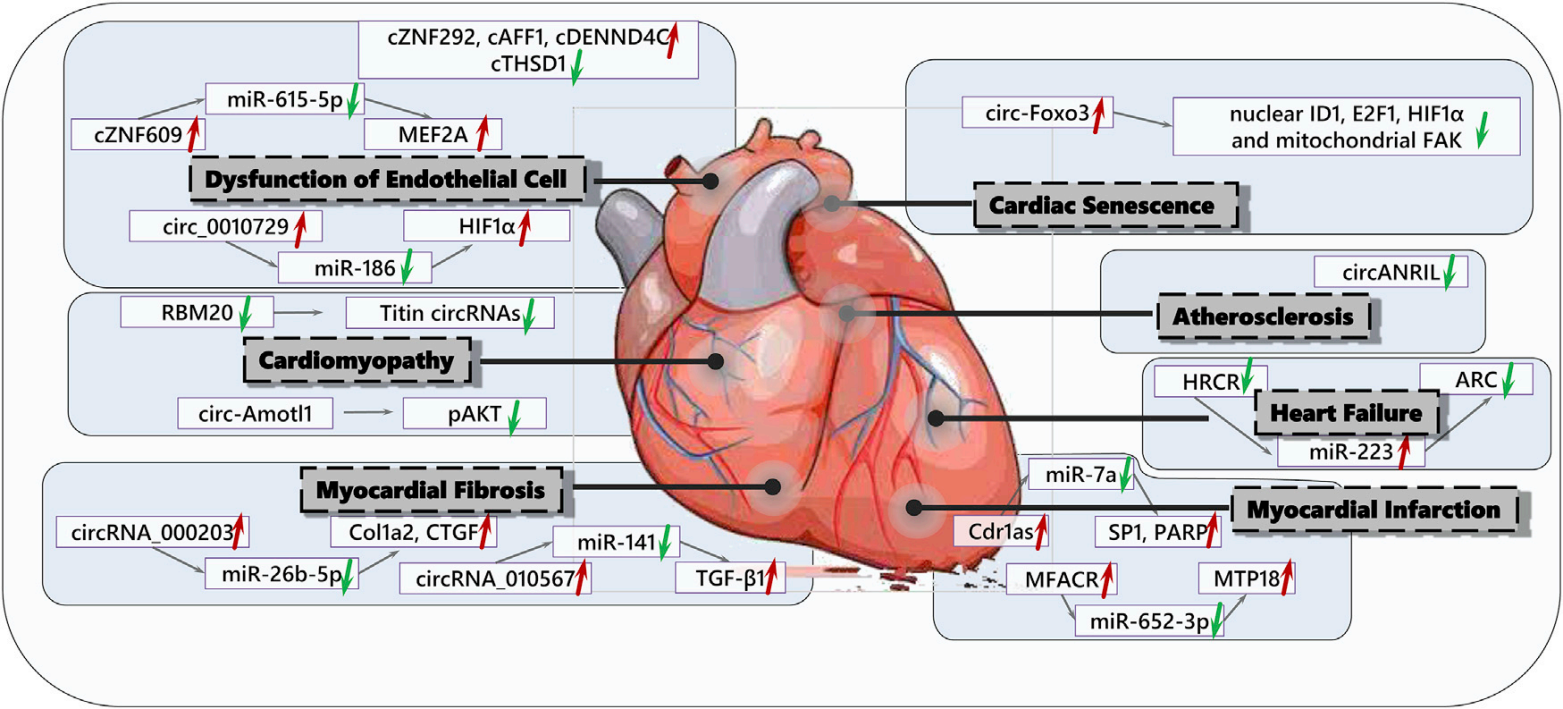
Circular RNAs and Their Targets in Cardiovascular Diseases

The roles of circRNAs in CVDs are summarized in Figure 3.

#### circRNAs in Cardiac Fibrosis

Cardiac fibrosis is characterized by excessive accumulation of extracellular matrix, leading to the damage of normal cardiac architecture and progressive cardiac dysfunction.^43^ Microarray analysis of myocardium from diabetic db/db mice and control db/m mice yielded 43 circRNAs that were differentially expressed (fold-change > 3.0; p < 0.05), 24 upregulated and 19 downregulated.^44^ Among them, circRNA_010567 was significantly upregulated in myocardium from diabetic db/db mice and Angiotensin II (AngII)-treated cardiac fibroblasts (CFs).^44^ Based on bioinformatics analysis, circRNA_010567 was predicted to sponge miR-141, which could target TGF-β1.^44^ The Pearson’s correlation further confirmed a negative correlation of circRNA_010567 and miR-141 in diabetic db/db mice.^44^ Suppression of circRNA_010567 was able to increase miR-141 and decrease transforming growth factor-β1 (TGF-β1) and inhibit fibrosis-associated protein expression in CFs, including Col I, Col III, and α-smooth muscle actin (α-SMA).^44^ Moreover, overexpression of circRNA_010567 increased fibrosis-associated protein expression by targeting the miR-141/TGF-β1 axis.^44^

Another similar microarray study was performed using pooled total RNA extracted from the myocardium of eight matched diabetic db/db mice and db/m control mice.^45^ circRNA_000203 was significantly increased in the myocardium of diabetic db/db mice and AngII-treated CFs.^45^ Overexpression of circRNA_000203 was able to increase Col1a2, Col3a1, and α-SMA in CFs.^45^ Mechanistically, miR-26b-5p was sponged by circRNA_000203 as confirmed by RNA pull-down and RT-PCR assay.^45^ Dual luciferase reporter assay further demonstrated that circRNA_000203 could block the interaction of miR-26b-5p with the 3′ UTR of Col1a2 and connective tissue growth factor (CTGF).^45^ Moreover, circRNA_000203 overexpression could inhibit the anti-fibrosis effect of miR-26b-5p in CFs.^45^

These studies suggest that circRNA_010567 and circRNA_000203 may act as novel contributors to cardiac fibrosis. However, their *in vivo* function in cardiac fibrosis and their effect in the differentiation of CFs to myofibroblasts remain largely unknown.

#### circRNAs in Myocardial Infarction

Myocardial infarction (MI) is a common manifestation of coronary artery disease (CAD) and represents a major cause of death among all CVDs.^46^ ciRS-7, also termed as Cdr1as, contains more than 70 conserved miR-7 target sites, thus reducing miR-7 activity and increasing target genes of miR-7.^18^ Expression of human Cdr1as in zebrafish had the similar effect of reducing miR-7, which impaired midbrain development.^47^ ciRS-7 and its sponged miR-7 have also been reported in cancer development.^48^, ^49^ Interestingly, Cdr1as and miR-7a were both increased in an MI mouse model and hypoxia-treated CMs,^50^ which may be due to the fact that Cdr1as could regulate miR-7 stability at posttranscriptional level.^27^ In mouse cardiac myocyte (MCM) cell line, Cdr1as overexpression could increase cell apoptosis, and this effect was inhibited by overexpression of miR-7a.^50^ Specificity protein 1 (SP1) and Poly (ADP-ribose) polymerase (PARP) were identified as target genes of miR-7a, and PARP and SP1 overexpression could attenuate the protective effect of miR-7a against cell apoptosis under hypoxia.^50^ Importantly, Cdr1as overexpression in mice could increase cardiac infarct size in association with increased expression of PARP and SP1, while miR-7a overexpression reversed these changes induced by Cdr1as overexpression.^50^ Thus, Cdr1as may contribute to MI by targeting the miR-7a/PARP-SP1 pathway.

Mitochondrial protein 18 kDa (MTP18) was increased in CMs subjected to anoxia and reoxygenation (A/R), which could lead to mitochondrial fission and cell apoptosis.^51^ Suppression of MTP18 decreased A/R-induced apoptosis in CMs and reduced cardiac infarct size in mice with ischemia and reperfusion (I/R) injury.^51^ MTP18 was identified as a target gene of miR-652-3p that was downregulated in CMs under A/R, while overexpression of miR-652-3p inhibited mitochondrial fission and CM apoptosis.^51^ To further identify circRNAs that regulate miR-652-3p, circRNAs from a circRNA online database were randomly screened and mm9_circ_016597 (also named mitochondrial fission and apoptosis-related circRNA, MFACR) was found to be increased in A/R and I/R models. Moreover, knockdown of MFACR could reduce mitochondrial fission and apoptosis *in vitro* and *in vivo*.^51^ Thus, MFACR may work as a sponge for miR-652-3p to increase MTP18 and mediate mitochondrial fission and CM apoptosis in MI.^51^

#### circRNAs in HF

HF is a multifactorial complex disease with high mortality and morbidity that is usually linked to functional and structural damage of ventricular filling or ejection of blood.^52^ A circRNA microarray analysis was used to determine dysregulated circRNAs in mouse myocardium with HF caused by MI.^53^ A total of 63 circRNAs were found to be differentially expressed in mouse myocardium 8 weeks post-MI (fold change ≥ 2.0, p ≤ 0.05): 29 upregulated and 34 downregulated.^53^ Interestingly, the expression of these circRNAs was not correlated with the expression of their host genes, indicating an independent regulation of circRNA formation versus transcription.^53^ To further identify potential miRNA targets, two circRNAs (circ_013216 and circ_010567) that were upregulated in HF as confirmed by qPCR were selected, and miRNA target prediction software TargetScan and miRanda were used to predict the circRNA and/or miRNA interaction. The potential miRNA targets of circ_013216 included miR-181a-3p, miR-486a-5p, and miR-486b-5p. The potential miRNA targets of circ_010567 included miR-124, miR-200a, and miR-141.^53^

Sustained and decompensated cardiac hypertrophy is an essential pathological process leading to HF. A miRNA microarray analysis was used to determine dysregulated miRNAs in isoproterenol (ISO)-induced cardiac hypertrophy and HF mouse model.^20^ As confirmed by qPCR and northern blot, miR-223-5p was proved to be consistently increased in ISO-induced HF and transverse aortic constriction (TAC)-induced HF mouse models, as well as in human failing heart samples.^20^ MiR-223 overexpression was sufficient to induce cardiac hypertrophy, while miR-223 inhibition blocked ISO-induced hypertrophy *in vivo*.^20^ Apoptosis repressor with CARD domain (ARC) was identified as a downstream target of miR-223, and ARC knockdown attenuated the inhibitory effect of miR-223 knockdown on hypertrophic response.^20^ One hundred circRNAs from the circRNA database were randomly screened, and mm9_circ_012559 named HRCR was found to be downregulated in ISO- and TAC-induced cardiac hypertrophy and HF models.^20^ RNAhybrid prediction, biotin-based pull-down assay, Ago2 immunoprecipitation, and fluorescence *in situ* hybridization (FISH) proved that HRCR directly bound to miR-223.^20^ Moreover, forced expression of HRCR in CMs and in mice attenuated hypertrophic responses *in vitro* and *in vivo*.^20^ Thus, enforced expression of HRCR may represent a novel therapy for cardiac hypertrophy and HF.

#### circRNAs in Dilated Cardiomyopathy

Dilated cardiomyopathy is a common myocardial disease usually having a molecular genetic basis. Patients with dilated cardiomyopathy progressively develop ventricular chamber enlargement and contractile dysfunction, leading to HF in the end stage.^54^ Loss of RNA-binding motif protein 20 (RBM20) may cause dilated cardiomyopathy most probably because it is necessary for normal splicing of many cardiac genes.^55^ Based on ribosomal-depleted RNA from human hearts, circRNA profiling was performed, which yielded multiple TTN circRNAs that were dynamically regulated in dilated cardiomyopathy, but not in hypertrophic cardiomyopathy (fold-change > 2.0, p < 0.05).^55^ RBM20-null mice completely lacked these TTN circRNAs and TTN circRNA production was also severely impaired in a cardiac sample from an RBM20 mutation carrier.^55^ Therefore, RBM20 may be important for circRNA formation, which indicates new mechanistic insights for dilated cardiomyopathy.^55^ Besides, the abundance of specific circRNAs from SLC8A1, CHD7, ATXN10, and DNAJC6 has been reported to be significantly regulated in dilated cardiomyopathy patients undergoing heart transplantation.^56^

#### circRNAs in Doxorubicin-Induced Cardiomyopathy

Doxorubicin (Dox)-induced cardiomyopathy is a well-known condition of myocardial dysfunction following chemotherapeutic treatment. circRNAs were compared between neonatal and mature postnatal human cardiac tissue samples based on circRNA microarray.^57^ Circ-Amotl1 was found to be preferentially expressed in neonatal cardiac tissues as compared to those from mature postnatal cardiac tissues. In CFs, circ-Amotl1 enhanced cell proliferation and survival while decreasing apoptosis.^57^ In primary isolated CMs and endothelial cell line YPEN, circ-Amotl1 increased cell survival while decreasing apoptosis. In these cells, circ-Amotl1 silence had opposite effect. Moreover, circ-Amotl1 overexpression in Dox-treated mice relieved the cumulative effect of Dox, as evidenced by reduced enlargement of left ventricle, abrogated fibrotic remodeling, and reduced apoptosis.^57^ Mechanistically, circ-Amotl1 could increase phospho-AKT (pAkt) in primary CMs and mouse myocardium. Circ-Amotl1 and pAKT co-localized with DAPI was detected, indicating that circ-Amotl1 may facilitate the nuclear translocation of pAKT. In support of this hypothesis, circ-Amotl1 knockdown repressed pAKT nuclear translocation. Furthermore, Triciribine, an AKT inhibitor, abolished the functional effect of circ-Amotl1 in CFs and endothelial cells.^57^ Thus, circ-Amotl1 may protect against Dox-induced cardiomyopathy by activating AKT phosphorylation and nuclear localization.

#### circRNAs in Cardiac Senescence

Aging is an independent risk factor for CVDs, which makes the heart more vulnerable to stress. Organismal aging is closely related to cellular senescence. Treatment of several cell lines with H_2_O_2_, including mouse embryonic fibroblasts (MEFs), mouse CFs (MCFs), NIH 3T3 fibroblasts, B16 cells, and primary CMs, caused significant cellular senescence with increased circ-Foxo3 expression.^22^ Moreover, circ-Foxo3 was upregulated in the myocardium from aged humans and mice.^22^ Circ-Foxo3 knockdown attenuated MEF senescence, while forced expression of circ-Foxo3 increased that.^22^ Circ-Foxo3 was mainly expressed in the cytoplasm, where it interacted with the anti-senescent proteins ID1 and E2F1 and the anti-stress proteins FAK and HIF1α.^22^ Circ-Foxo3 was pulled down by antibodies against ID1, E2F1, FAK, and HIF1α, while the linear Foxo3 mRNA was not.^22^ Increased circ-Foxo3 in the cytoplasm decreased the expression level of these proteins in the nucleus and mitochondria and thus blocked their anti-senescent and anti-stress functions.^22^ Moreover, silencing circ-Foxo3 could relieve Dox-induced cardiac premature senescence in mice.^22^ Thus, reducing circ-Foxo3 may have anti-senescent and protective effect for the heart.

#### circRNAs in Atherosclerosis

Atherosclerosis is initiated by lipid deposition in the subendothelial layer of arterial wall and features inflammatory infiltration of macrophages, dendritic cells, and activated T cells.^58^ Circular antisense ncRNA in the INK4 locus (circANRIL), transcribed at a locus of atherosclerotic CVD on chromosome 9p21, may confer atheroprotective function.^58^ High expression level of circANRIL in human vascular tissues was associated with less severity of CAD. Mechanistically, circANRIL was proved to bind to pescadillo homolog 1 (PES1), an essential 60S-pre-ribosomal assembly factor, possibly leading to impaired exonuclease-mediated pre-ribosomal RNA processing and ribosomal biogenesis in vascular smooth muscle cells and macrophages. In turn, circANRIL induced p53 activation, leading to increased apoptosis while reduced proliferation of vascular smooth muscle cells and macrophages.^58^ Thus, circANRIL may protect against atherosclerosis by inhibiting overproliferation of cells in atherosclerotic plaque.

#### circRNAs as Regulators of Other Functions Related to CVDs

Endothelial dysfunction plays a critical role in the pathogenesis of many CVDs, including atherosclerosis, hypertension, thrombus formation, and diabetic cardiovascular complication.^59^ A total of 7,388 endothelial circRNAs have first been identified in ribosomal RNA-depleted RNA of human umbilical vein endothelial cells (HUVECs) based on PYTHON scripts provided by circBase.^59^ As hypoxia is a key stimulus for angiogenesis, HUVECs were exposed to hypoxia and the hypoxia mimic COCl_2_.^59^ As confirmed by qPCR, cZNF292, cAFF1, and cDENND4C were significantly increased, while cTHSD1 was decreased in hypoxia-treated HUVECs.^59^ circRNAs cZNF292, cDENND4C, and cTHSD1 correlated with host-gene expression under hypoxia, whereas cAFF1 was independent of hypoxia-induced transcriptional change.^59^ Moreover, silencing cZNF292 was able to decrease spheroid sprouting and tube formation in Matrigel assays and inhibit endothelial cell proliferation.^59^ These data provide strong evidence that circRNAs can also be expressed in endothelial cells and exert biological function in angiogenesis.

Based on circRNA microarray analysis and qPCR, hsa_circ_0010729 was also found to be significantly increased in hypoxia-treated HUVECs.^60^ Circ_0010729 knockdown significantly inhibited the proliferation and migration while enhancing the apoptosis of HUVECs.^60^ Bioinformatics analysis and luciferase reporter assay further demonstrated that the co-expression of circ_0010729 with HIF-1α was negatively correlated with miR-186.^60^ Importantly, miR-186 inhibitor fully reversed the function of circ_0010729 knockdown on HUVECs.^60^ Thus, circ_0010729 may regulate vascular endothelial cell proliferation and apoptosis via targeting miR-186/HIF-1α pathway.

In addition to hypoxic stress, hyperglycemia is also a trigger for endothelial dysfunction. circRNA-ZNF609 (cZNF609), one of the abundantly expressed circRNAs in endothelial cells, was significantly upregulated in high glucose-treated HUVECs and also in the retina of a murine model of diabetic retinopathy.^61^ Silencing cZNF609 increased endothelial cell viability, proliferation, migration, and tube formation at baseline and partially reduced oxidative or hypoxic stress-induced cell apoptosis in HUVECs.^61^ miR-615-5p was predicated to be sponged by cZNF609, and miR-615-5p/cZNF609 interaction was proved to control endothelial cell function.^61^ In addition, MEF2A was a target gene of miR-615-5p and MEF2A overexpression fully rescued cZNF609 silencing-mediated effect on endothelial cell migration, tube formation, and apoptosis.^61^ Moreover, cZNF609 expression was determined in clinical samples from diabetic patients. The expression of cZNF609 in the fibrovascular membranes of diabetic patients was higher than that in the idiopathic epiretinal membranes of non-diabetic patients.^61^ Meanwhile, plasma cZNF609 level was significantly elevated in diabetic patients, whereas plasma miR-615-5p was decreased.^61^ Thus, intervention of cZNF609 may be a potential therapeutic strategy for endothelial dysfunction and vascular disease.

### Circulating circRNAs as Biomarkers for CVDs

Many circRNAs are expressed in a tissue- and stage- specific manner and therefore their dysregulation may reflect a dynamic disease state.^62^ Due to its circular structure, most circRNAs are resistant to RNase R digestion, making them highly stable in plasma, serum, or other biofluids.^63^ Moreover, circRNAs can be enriched in circulating extracellular vesicles.^64^, ^65^ The unusual stability and expression specificity make circRNAs promising biomarkers for CVDs. The studies about circulating circRNAs as potential biomarkers for CVDs are summarized in Table 2.

**Table 2:** Circulating circRNAs as Biomarkers for Cardiovascular Diseases

Diseases	circRNAs	Sources	Regulation	No. of Samples	Potential Use	Methods	Species	Reference
Coronary artery disease	24 dysregulated	plasma	18 upregulated, 6 downregulated	3 CAD and 3 control	–	microarray	human	^66^
	circ_0124644, circ_0098964	blood	upregulated	1st cohort: 12 CAD and 12 control 2nd cohort: 30 CAD and 30 control 3rd cohort: 137 CAD and 115 control	diagnostic	microarray and qRT-PCR	human	^67^
Myocardial infarction	MICRA	blood	downregulated	642 AMI from two independent cohorts	prognostic	qRT-PCR	human	^68^
Hypertension	circ_0005870	plasma	downregulated	54 hypertension and 54 healthy	–	microarray and qRT-PCR	human	^69^
Carotid plaque rupture	ratio of circRNA-284: miRNA-221	serum	upregulated	1st cohort: 24 asymptomatic and 17 acutely symptomatic 2nd cohort: 47 asymptomatic, 41 acutely symptomatic, and 24 symptomatic	diagnostic	qRT-PCR	human	^70^
Pre-diabetes and type 2 diabetes mellitus	circ_0054633	blood	upregulated	1st cohort: 6 T2DM and 6 healthy; 2nd cohort: 20 T2DM, 20 pre-diabetes, and 20 healthy 3rd cohort: 64 T2DM, 63 pre-diabetes, and 60 healthy	diagnostic	microarray and qRT-PCR	human	^71^
Coronary artery disease and type 2 diabetes mellitus	circ_11783-2	blood	downregulated	1st cohort: 6 healthy, 6 CAD, 6 T2DM, and 6 CAD combined with T2DM; 2nd cohort: 20 healthy, 20 T2DM, and 20 CAD 3rd cohort: 60 healthy, 64 T2DM, and 81 CAD	diagnostic	microarray and qRT-PCR	human	^71^
Chronic thromboembolic pulmonary hypertension	351 dysregulated	blood	122 upregulated, 229 downregulated	5 healthy and 5 CTEPH	–	microarray	human	^72^

CAD, coronary artery disease; MICRA, myocardial infarction-associated circular RNA; AMI, acute myocardial infarction; T2DM, type 2 diabetes mellitus; CTEPH, chronic thromboembolic pulmonary hypertension.

#### Circulating circRNAs as Biomarkers for CAD

Plasma circRNA profiling was performed in three CAD patients versus three control subjects.^66^ A total of 18 circRNAs were found to be upregulated in CAD patients, while six were downregulated (fold change ≥ 1.5, p < 0.05). Meanwhile, plasma miR-221, miR-155, and miR-130a were detected to be downregulated in an independent population of 648 CAD patients versus 284 control subjects as determined by qPCR. Based on miRanda database, miR-130a-3p-mediated circRNA-mRNA competing endogenous RNA (ceRNA) network was constructed.^66^ This network was composed of nine circRNAs (hsa_circ_0089378, hsa_circ_0083357, hsa_circ_0082824, hsa_circ_0068942, hsa_circ_0057576, hsa_circ_0054537, hsa_circ_0051172, hsa_circ_0032970, and hsa_circ_0006323) and one mRNA (transient receptor potential cation channel subfamily M member 3 [TRPM3]).^66^ However, these results were based on bioinformatics analysis and did not provide a specific biomarker of CAD.

To identify specific biomarkers for CAD, peripheral blood circRNAs were analyzed using circRNA microarrays on samples from 12 CAD patients and 12 control individuals.^67^ Five upregulated circRNAs with the highest fold change (hsa_circ_0082081, hsa_circ_0113854, hsa_circ_0124644, hsa_circ_0098964, and hsa_circ_5974-1) were further validated in 30 CAD patients and 30 control individuals using qPCR.^67^ To determine the diagnostic value of these circulating circRNAs, receiver operating characteristic curve (ROC) analysis was performed showing that the area under curves (AUCs) of these circRNAs were 0.660, 0.689, 0.872, 0.820, and 0.743, respectively.^67^ As hsa_circ_0124644 had the highest AUC among these five circRNAs, its diagnostic value was further validated in an independent cohort consisting of 137 CAD patients and 115 control individuals. As determined by ROC analysis, the AUC of hsa_circ_0124644 was 0.769, and the sensitivity and specificity were 0.861 and 0.626, respectively.^67^ After adjusting for CAD risk factors including smoking, hypertension, diabetes mellitus, low-density lipoprotein (LDL), and total cholesterol (TC), the AUC of hsa_circ_0124644 increased to 0.804, with a sensitivity and specificity of 0.759 and 0.704, respectively. Combining hsa_circ_0124644 with hsa_circ_0098964, the AUC increased to 0.811, and the sensitivity and specificity increased to 0.825 and 0.730, respectively.^67^ Again, after adjustment for CAD risk factors, the AUC increased to 0.843, with a sensitivity and specificity of 0.832 and 0.696, respectively.^67^ Thus, the combination of hsa_circ_0124644 and hsa_circ_0098964 may serve as promising diagnostic biomarker of CAD. Future studies are warranted to determine whether and how circulating circRNAs correlate with the extent of CAD, such as plaque size, plaque complexity, and arterial calcification.

#### Circulating circRNAs as Biomarkers for MI

Outcome prediction after MI is challenging and novel prognostic biomarkers are highly needed.^68^ A total of 642 acute MI patients were enrolled: 409 from the Luxembourg Acute Myocardial Infarction Registry and 233 from the German LIFE-Leipzig Heart Study.^68^ MI-associated circRNA (MICRA) was identified based on an *in silico* approach and further validated by qPCR.^68^ MICRA contains 874 nucleotides and derives from exon 1 of the zinc finger protein 609 (ZNF609) gene located on chromosome 15q22.^68^ As assessed by qPCR, blood MICRA level measured at reperfusion was significantly lower in MI patients as compared to healthy controls.^68^ In both univariate and multivariate logistic regression analyses, MICRA showed strong predictive value of left ventricular dysfunction at 4 months after MI.^68^ Moreover, MICRA was demonstrated to help improve risk stratification for MI patients.^68^ Further studies are needed to confirm the predictive value of MICRA in combination or comparison with other markers of myocardial injury such as cardiac troponin (cTnT and cTnI) for MI patients.

#### Circulating circRNAs as Biomarkers for Hypertension

Hypertension is the most common risk factor for CVDs. Plasma circRNAs were profiled with circRNA microarrays in five hypertension patients and five healthy controls.^69^ Four circRNAs including hsa_circ_0000437, hsa_circ_0008139, hsa_circ_0005870, and hsa_circ_0040809 showed significant difference (fold-change > 2.0, p < 0.05), and hsa_circ_0005870 was further validated by qPCR to be significantly downregulated in hypertension patients.^69^ The gene ontology (GO) terms of hsa_circ_0005870 suggested a strong relationship with the biological process of RNA polymerase activity, DNA metabolic process, and cellular response to stress.^69^ The Kyoto Encyclopedia of Genes and Genomes (KEGG) pathway analysis of hsa_circ_0005870 indicated a strong relationship with the biological process of TGF-β signaling pathway, glycosphingolipid biosynthesis-globo series, and other types of O-glycan biosynthesis.^69^ These findings showed that plasma circ_0005870 was significantly decreased in hypertension patients, which requires further investigation for its potential use as biomarker for hypertension.

#### Circulating circRNAs as Biomarkers for Carotid Plaque Rupture

Carotid plaque rupture is a major cause of cardiovascular events such as stroke. Novel biomarkers for carotid plaque rupture may be useful in identifying at-risk patients. Serum levels of miR-221, miR-222, miR-145, and circRNA-284 were measured in 24 asymptomatic patients and 17 acutely symptomatic patients undergoing carotid endarterectomy.^70^ Serum miR-221 was significantly downregulated in the acutely symptomatic patient group as compared with the asymptomatic group.^70^ As circRNA-284 is a potential inhibitor of miR-221, the ratio of serum circRNA-284:miR-221 was further determined in asymptomatic and acutely symptomatic patients, although circRNA-284 tended to increase but without statistical significance in acutely symptomatic patients.^70^ Interestingly, the ratio of serum circRNA-284:miR-221 was significantly increased in the acutely symptomatic patient group, which was further confirmed in a validation cohort. Thus, the ratio of serum circRNA-284:miR-221 showed favorable characteristics as a biomarker indicative of carotid plaque rupture and stroke.^70^

#### Circulating circRNAs as Biomarkers for Pre-diabetes and Type 2 Diabetes Mellitus

Type 2 diabetes mellitus (T2DM) is an increasing public health problem and cardiovascular complications are a major contributor to morbidity and mortality in T2DM patients.^71^ However, early diagnosis methods of pre-diabetes and T2DM are still lacking. Peripheral blood circRNAs were profiled by human circRNA array between six T2DM patients and six control individuals and were further validated by qPCR.^71^ The expression levels of hsa_circ_0054633 and hsa_circ_0068087 were incrementally increased from the control group to the pre-diabetes group to the T2DM group (n = 20 per group).^71^ The AUCs of hsa_circ_0054633 and hsa_circ_0068087 for the diagnosis of pre-diabetes were 0.747 and 0.692, respectively.^71^ The AUCs of hsa_circ_0054633 and hsa_circ_0068087 for the diagnosis of T2DM were 0.720 and 0.717, respectively.^71^ In another cohort including 60 controls, 63 pre-diabetes, and 64 T2DM, hsa_circ_0054633 with a higher AUC was further analyzed to validate its potential as a diagnostic biomarker for pre-diabetes and T2DM. It was found that hsa_circ_0054633 increased 1.8-fold from controls to pre-diabetes, and increased 1.7-fold from pre-diabetes to T2DM.^71^ After adjusting for the risk factors of T2DM including smoking, hypertension, body mass index (BMI), TC, triglycerides (TG), high-density lipoprotein (HDL), and LDL, the AUCs of hsa_circ_0054633 for the diagnosis of pre-diabetes and T2DM increased from 0.751 to 0.841 and from 0.793 to 0.834, respectively.^71^ Thus, circulating hsa_circ_0054633 may serve as a novel diagnostic biomarker of pre-diabetes and T2DM.

In addition, hsa_circ_11783-2 level in peripheral blood was found to be correlated with both T2DM and CAD.^71^ Based on the human circRNA array, circRNAs were profiled in peripheral blood samples from six healthy controls, six CAD patients, six T2DM patients, and six CAD combined with T2DM patients.^71^ A total of 40 circRNAs were differentially expressed between all three experimental groups and the control group, and selected circRNAs were validated by qPCR in another cohort consisting of 20 healthy controls, 20 T2DM patients, and 20 CAD patients. Three circRNAs including hsa_circ_11806-28, hsa_circ_6510-1, and hsa_circ_11783-2 were significantly lower in both the T2DM group and the CAD group compared with the control group.^71^ After introducing the common risk factors of CAD and T2DM (smoking, hypertension, TC, TG, HDL, and LDL), a stronger correlation of hsa_circ_11783-2 with CAD and T2DM was observed as compared to hsa_circ_11806-28 and hsa_circ_6510-1.^71^ In another cohort with larger sample size, the adjusted odds ratio of hsa_circ_11783-2 was 0.688 in the CAD group and 0.723 in the T2DM group.^71^ These data indicated that blood hsa_circ_11783-2 was closely related to both CAD and T2DM, though its role as a possible diagnostic biomarker should be further investigated in the future.

#### Circulating circRNAs as Biomarkers for Pulmonary Hypertension

Pulmonary hypertension (PH) is defined by the elevation of pulmonary arterial pressure related to different etiologies. Chronic thromboembolic pulmonary hypertension (CTEPH) is a rare but severe complication of pulmonary embolism. Early diagnosis of CTEPH may help make early clinical decision, thus improving prognosis for patients. Blood circRNAs were profiled by Agilent circRNA chip using samples from five CTEPH patients and five healthy controls.^72^ A total of 351 circRNAs were found to be dysregulated (p < 0.05). Among them, 122 circRNAs were upregulated, and these circRNAs were enriched in the purine ribonucleotide biosynthetic process.^72^ In addition, 229 circRNAs were downregulated, and these circRNAs were enriched in cellular response to stress and DNA damage stimulus, DNA repair, posttranscriptional regulation of gene expression, and mRNA metabolic process.^72^ Moreover, hsa_circ_0002062 and hsa_circ_0022342 were predicted to be particularly important as they regulated 761 (e.g., miR-942-5p) and 453 (e.g., miR-940) miRNAs, respectively.^72^ Target genes of miR-942-5p were mainly enriched in cancer-related pathways, while those of miR-940 were enriched in the ErbB signaling pathway. Both pathways are of critical importance in the pathogenesis of CTEPH.^72^ However, a specific blood circRNA biomarker of CTEPH still needs to be identified.

### Future Perspectives

Due to the advance of bioinformatics tools and RNA-sequencing (RNA-Seq) techniques, circRNAs are recognized as stable, abundant, and novel players in the regulation of gene expression.^73^ However, the functional roles of circRNAs are still far from understood as compared with the progress made in the field of other ncRNAs such as miRNAs and lncRNAs. First, most available studies about circRNAs only reveal associations with a disease state in human or a disease animal model. The vast majority of circRNAs have not yet been studied, and their functions remain unknown. Additionally, it is worth noting that some circRNAs have been reported to be involved in different CVDs. For example, circ_010567 was upregulated in diabetic mouse myocardium and promoted cardiac fibrosis.^44^ Meanwhile, circ_010567 was found to be upregulated in HF mouse myocardium caused by MI.^53^ Thus, the relative contribution of the same circRNA(s) to different CVDs deserve further investigation. Second, the strategies available for deeply studying circRNAs have not yet been well developed. One is to perform the microarray or sequencing analysis for identifying candidate circRNAs in a disease model. Another is to search candidate circRNAs based on the available databases and/or published literatures. The candidate circRNAs were then selected for qPCR validation, circularization confirmation, and functional studies. However, most studies only identify circRNAs as sponges of miRNA while it is currently accepted that this may not be the major biological function of circRNAs. Notably, it was recently reported that circRNAs can be translated, and the role of translated circRNAs in CVDs needs further investigation.^74^, ^75^ Third, comparison of circRNAs in human and mouse datasets suggested that 15% of the circRNAs used the precisely conserved splice sites in mouse and/or human orthologous genes.^15^, ^76^ In heart, approximately 30% of the circRNAs are conserved between mouse and rat, and 10% being conserved across human, mouse, and rat.^16^ Therefore, it is currently recommended to study the sequence-conserved circRNAs so that the discoveries from animal models could prospectively be translated into human disease research. Projects exploring the clinical application of circRNAs in CVDs will be needed to promote translational research.

circRNAs are stable in the circulation, and their dynamic changes may reflect disease stage, including CVDs. In this review, we also summarized the advance in studies identifying circulating circRNAs as potential biomarkers for CVDs. However, it should be noted that the use of circulating circRNAs as biomarkers for CVDs is still in its infancy. Most studies are limited by small sample size, a lack of independent validation cohorts, and limited analysis of correlation with disease characteristics. Proper endogenous control for data normalization is also unknown. In the future, the value of circulating circRNAs should be evaluated based on its incorporation into the current clinical models. Also, the guidelines for determining circulating circRNAs as biomarkers should be provided by the community so that the data between different groups could be compared.

In conclusion, circRNAs have emerged as novel players in the cardiovascular field. In the future, RNA sequencing and loss- and gain-of-function analysis in relevant models are warranted to further reveal the link of circRNAs with CVDs and finally pave their road from bench to beside.

## Author Contributions

All authors contributed to the drafting, editing, and revision of the manuscript and read and approved the final manuscript.

## Conflicts of Interest

S.D. is a founder and on the Scientific Advisory Board of Dyrnamix.
